# t(15;21) translocations leading to the concurrent downregulation of *RUNX1* and its transcription factor partner genes *SIN3A* and *TCF12* in myeloid disorders

**DOI:** 10.1186/s12943-015-0484-0

**Published:** 2015-12-16

**Authors:** Alberto L’Abbate, Doron Tolomeo, Francesca De Astis, Angelo Lonoce, Crocifissa Lo Cunsolo, Dominique Mühlematter, Jacqueline Schoumans, Peter Vandenberghe, Achilles Van Hoof, Orazio Palumbo, Massimo Carella, Tommaso Mazza, Clelia Tiziana Storlazzi

**Affiliations:** Department of Biology, University of Bari, Bari, Italy; UO Anatomia Patologica, Ospedale S. Martino, Belluno, Italy; Unité de génétique du cancer, Service de génétique médicale, Centre Hospitalier Universitaire Vaudois CHUV, Lausanne, Switzerland; Center for Human Genetics and Department of Hematology, University Hospital Leuven and KU Leuven, Leuven, Belgium; Department of Haematology, AZ Sint-Jan AV, Brugge, Belgium; Medical Genetics Unit, IRCCS Casa Sollievo della Sofferenza Hospital, San Giovanni Rotondo, Italy; IRCCS Casa Sollievo della Sofferenza, Mendel Institute, San Giovanni Rotondo, Italy

**Keywords:** Haploinsufficiency, Tumor suppressor genes, AML, MDS

## Abstract

Through a combined approach integrating RNA-Seq, SNP-array, FISH and PCR techniques, we identified two novel t(15;21) translocations leading to the inactivation of *RUNX1* and its partners *SIN3A* and *TCF12*. One is a complex t(15;21)(q24;q22), with both breakpoints mapped at the nucleotide level, joining *RUNX1* to *SIN3A* and *UBL7-AS1* in a patient with myelodysplasia. The other is a recurrent t(15;21)(q21;q22), juxtaposing *RUNX1* and *TCF12,* with an opposite transcriptional orientation, in three myeloid leukemia cases. Since our transcriptome analysis indicated a significant number of differentially expressed genes associated with both translocations, we speculate an important pathogenetic role for these alterations involving *RUNX1*.

**Electronic supplementary material:**

The online version of this article (doi:10.1186/s12943-015-0484-0) contains supplementary material, which is available to authorized users.

## Main text

Translocations involving *RUNX1* are known to decrease the function of the encoded protein in myelodysplastic syndromes (MDS) and acute myeloid leukemia (AML) [1]. For those involving chromosome 15, *SV2B* was the only *RUNX1* partner gene identified in AML [2].

We report on two novel t(15;21) alterations leading to the concurrent disruption of *RUNX1* and *SIN3A* or *TCF12* (Additional file 1: Table S1). Another interrupted gene is the *UBL7-AS1* long noncoding RNA gene.

**Fig. 1:**
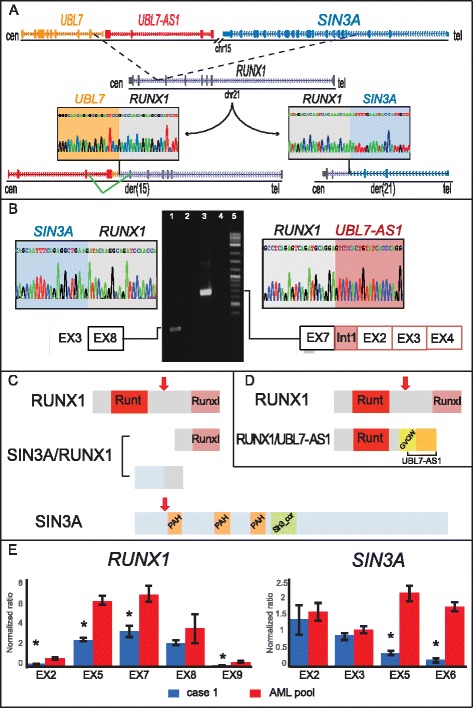
t(15;21)(q24;q22) translocation in case 1. **a** *Genomic breakpoints on chromosomes 15 and 21 in case 1*: Top, schematic representation of wild-type chromosomes (black lines) and involved genes (exons are shown as rectangles and introns as lines connecting exons, with arrowheads indicating the direction of transcription). The dashed black lines indicate the breakpoints within all genes. Bottom, partial chromatograms indicate fusion sequences on der(15) (left) [GenBank: KT336107] and der(21) (right) [GenBank: KT336106]. The splicing event creating the runt-related transcription factor 1 (*RUNX1*)/*UBL7-AS1* fusion transcript is indicated in green. **b** RUNX1 *fusion transcripts*: reverse transcription polymerase chain reaction (RT-PCR) products corresponding to *SIN3A*/*RUNX1* [GenBank: KT336104] and *RUNX1*/*UBL7-AS1* [GenBank: KT336105] fusion transcripts (lanes 1 and 3, respectively) in case 1 are shown in the middle of the panel. Lanes 2 and 4: negative normal bone marrow samples. Lane 5: 2-log DNA ladder (New England Biolabs, Milan, Italy). Partial chromatograms (top) and structure (bottom) of *SIN3A*/*RUNX1* and *RUNX1*/*UBL7-AS1* PCR products are on the left and on the right, respectively. **c**, **d** *RUNX1 chimeric proteins*: both panels show in silico translation (ORFfinder and BlastP) of both wild-type and chimeric RUNX1 and SIN3A proteins. Arrows indicate the truncation breakpoints of wild-type proteins. **e** *Evaluation of* RUNX1 *and* SIN3A *expression levels in case 1:* exon-specific reverse transcription quantitative PCR analysis of *RUNX1* (left) and *SIN3A* (right) was performed in case 1 vs a control pooled sample of patients with acute myeloid leukemia. Asterisks indicate statistically significant results (*P* < .05)

In case 1, FISH experiments (Additional file 2: Table S2) mapped the breakpoint on der(21) within intron 7 of *RUNX1* (Additional file 3: Figure S1A-C). Moreover, SNP array analysis identified a 908-kb deletion near the 15q breakpoint on der(15) (Additional file 4: Table S3 and Additional file 3: Figure S1D-F). Genomic PCR revealed that intron 7 of *RUNX1* (chr21:36194775) was joined at intron 3 of *SIN3A* (chr15:75708434) (Fig. 1a and Additional file 5: Table S4) on der(21). Furthermore, intron 7 of *RUNX1* (chr21:36194861) was fused to the inverted sequence of *UBL7* (NM_032907.4) at intron 1 (chr15:74751664) on der(15), suggesting that a submicroscopic inversion accompanying the translocation led to the juxtaposition of *RUNX1* and *UBL7-AS1* with the same transcriptional orientation (Fig. 1a). ChimeraScan analysis of RNA-Seq data identified the fusion of *SIN3A* (exon 3; NM_015477) to *RUNX1* (exon 8; NM_001754) and of *RUNX1* (exon 7; NM_001754) to *UBL7-AS1* (intron 1; NR_038449.1). Both chimeric transcripts were validated by RT-PCR (Fig. 1b and Additional file 6: Table S5). In silico translation of the in-frame 5′-*SIN3A/*3′-*RUNX1* showed two ORFs of 171 and 163 amino acids, respectively, the first one retaining the transactivation domain of RUNX1 (Fig. 1c). The out-of-frame 5′-*RUNX1/*3′-*UBL7-AS1* encoded a protein of 373 amino acids, showing the substitution of RunxI with a GVQW putative binding domain (Fig. 1d). Thus, both chimeric *SIN3A*/*RUNX1* and *RUNX1*/*UBL7-AS1* encoded for truncated SIN3A and RUNX1 proteins. Interestingly, RT-qPCR revealed that the full-length *RUNX1* (NM_001754) and the 3′ portion of *SIN3A* (NM_015477) were downregulated (Fig. 1e), whereas *UBL7-AS1* was overexpressed (Additional file 7: Figure S2). Further, we evaluated the molecular impact of these fusion transcripts through a differential expression analysis, using the control datasets of 19 AML cases with normal karyotype from the The Cancer Genome Atlas data bank (http://cancergenome.nih.gov/). The results obtained by the QIAGEN’s Ingenuity® Pathway Analysis (IPA®, QIAGEN Redwood City) [3], following the Partek Genomics Suite 6.6 (Partek Inc., St. Louis, MO, USA), indicated a significant number of differentially expressed genes within the AML pathway. The cell proliferation and myeloid differentiation pathways were significantly activated, whereas apoptosis exhibited reduced activity (Additional file 8: Figure S3 and Additional file 9: Table S6).

The results obtained in case 1 clearly suggest a role as a tumor suppressor (TS) gene not only for *RUNX1* (the shorter RUNX1 encoded by the chimeric *RUNX1*/*UBL7-AS1* should behave as a dominant negative mutant of the wild-type RUNX1), but also for *SIN3A.* We speculate that the inactivation of both proteins should have led to an abnormal activation of *RUNX1*/*SIN3A* target genes, leading to myelodysplasia. Even if haploinsufficiency was never reported for *SIN3A*, its role as a TS has been described in other tumors [4]. Notably, the SIN3A corepressor was known to interact with RUNX1 [5], leading to the transcriptional inactivation of their target genes [6].

**Fig. 2:**
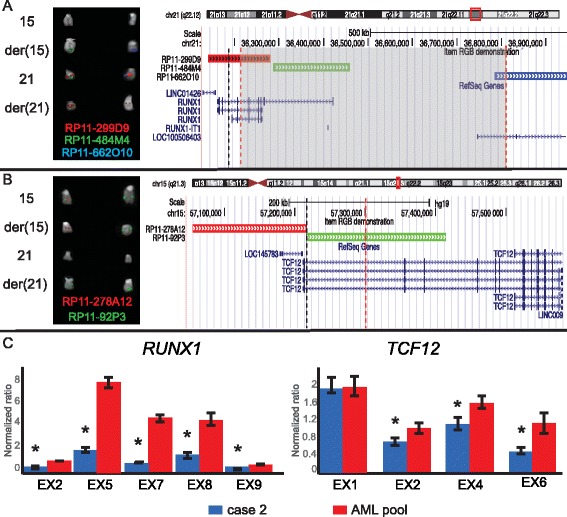
t(15;21)(q21;q22) translocation in cases 2, 3, and 4. **a**, **b** *Breakpoints on chromosomes 21 and 15*: partial karyotypes showing fluorescence in situ hybridization (FISH) results that allowed the mapping of t(15;21) translocation breakpoints on der(21) (a) and der(15) (b), using the consistently colored probes listed for cases 2 (first column) and 4 (second column). RP11-662O10 was used only in case 4. Cases 2 and 3 shared the same breakpoints (data not shown). On the right, the map of the BAC probes used in the FISH experiments, according to GRCh37/hg19, and identifying both translocation and deletion breakpoints, is shown. The black and orange dashed lines indicate the breakpoints in cases 2 and 4, respectively. The grey rectangle encompasses the deleted region flanking the translocation breakpoint in case 4. **c** *Evaluation of RUNX1 and TCF12 expression levels*: exon-specific reverse transcription quantitative polymerase chain reaction results of runt-related transcription factor 1 (*RUNX1*; left) and transcription factor 12 (*TCF12*; right) in case 2. Asterisks indicate statistically significant results (*P* < .05)

In cases 2 and 3, FISH indicated that *RUNX1* was interrupted within intron 7. Additionally, in case 4, a 600-kb deletion removed the 5′ portion of *RUNX1* starting from intron 6 (Fig. 2a and Additional file 1: Table S1). In all cases, *RUNX1* was joined with an opposite transcriptional orientation to intron 3 of *TCF12* (NM_003205), a basic helix-loop-helix transcription factor (Fig. 2b) fused with *MLL* in MDS [7] and recurrently mutated in myeloproliferative disorders [8]. Notably, in case 2, RT-qPCR indicated the downregulation of both *RUNX1* and *TCF12* (Fig. 2c) and IPA analysis disclosed significant deregulation of the AML pathway. The number of altered pathways was slightly higher than in case 1 (146 vs. 136) and mostly overlapped the previously described categories. Particularly, *RUNX1* was shown to control many differentially expressed genes involved in cell cycle regulation, inflammatory response, and transcription regulation (Additional file 8: Figure S3 and Additional file 9: Table S6).

We thus suggest a TS role for *TCF12* in myeloid disorders, as already described in colon carcinoma [9]. The concurrent inactivation of *RUNX1* and *TCF12* should mimic the same leukemogenic effect of the t(8;21) RUNX1/CBFA2T1 fusion protein. E proteins, like TCF12, are inactivated through their interaction with the domain TAFH of CBFA2T1, leading to the inhibition of the p300/CBP histone acetyltransferase recruitment at their target genes’ promoters and consequently to the lack of activation of genes with E-box promoters [10].

To summarize, we here identified three novel *RUNX1* partner genes, including two transcription factors and a long noncoding RNA, in 2 t(15;21) translocations. Both of the t(15;21) translocations resulted in the concurrent inactivation of *RUNX1* and one related transcription factor (*SIN3A* or *TCF12*), leading to the potential haploinsufficiency of both involved genes. Moreover, the IPA analyses clearly indicated that the AML pathway was significantly deregulated in our samples, and showed that *RUNX1*, *SIN3A*, or *TCF12* have a crucial impact on differentially expressed genes. The analysis of additional cases harboring t(15;21) translocations will be helpful to better understand the pathogenetic impact of these alterations in myeloid neoplasms.

### Ethics approval

This study was performed in agreement with the Declaration of Helsinki, and approved by the Ethical Committee at the “S. Martino” hospital, Belluno (Italy); Centre Hospitalier Universitaire Vaudois CHUV, Lausanne (Switzerland); University Hospital Leuven, (Belgium).

### Consent for publication

Written informed consent was obtained from the patients for publication of this letter.

## Additional files

Additional file 1: Table S1. Cytogenetic and clinical features of the patients included in the study. (DOCX 8 kb) Additional file 2: Table S2. FISH results obtained with BAC and fosmid probes in all the cases included in the study. (DOCX 125 kb) Additional file 3: Figure S1. FISH mapping of the t(15;21) translocation breakpoints in case 1. (A, B) Merged pseudocolored fluorescence in situ hybridization (FISH) results obtained with consistently colored BAC and fosmid probes spanning runt-related transcription factor 1. (C) Map of the probes used in the FISH experiments in panels A and B. The black dashed line indicates the breakpoint within the gene. (D, E) FISH results obtained with BAC and fosmid probes used to map the proximal (D) and distal breakpoints (E) on chromosome 15. (F) Map of the probes used in the FISH experiments, identifying both translocation and deletions breakpoints. Black dashed lines indicate the breakpoints; the grey rectangle encompasses the deleted region flanking the translocation breakpoint. (EPS 3237 kb) Additional file 4: Table S3. SNP array results [EMBL: E-MTAB-3782] obtained in case 1. (DOCX 123 kb) Additional file 5: Table S4. List of primers used for RT-PCR, RT-qPCR and Sanger sequencing. (DOC 91 kb) Additional file 6: Table S5. Case 1 Chimerascan output: in bold the fusion genes validated by RT-PCR and Sanger sequencing. Case 2 Chimerascan output. (XLSX 309 kb) Additional file 7: Figure S2. Expression pattern analysis of *UBL7-AS1* in case 1. Exon-specific reverse transcription quantitative polymerase chain reaction analysis of *UBL7-AS1* in case 1 vs a control pooled sample of patients with acute myeloid leukemia. Asterisks indicate statistically significant results (*P* < .05). (EPS 246 kb) Additional file 8: Figure S3. The IPA acute myeloid leukemia signaling pathway for cases 1 and 2. Orange and blue glyphs indicate genes with predicted enhanced or reduced activity, respectively. Green symbols represent downregulated genes. Equally, orange and blue toothed wheels stand for activated or inhibited biological processes. Case 1 differs from case 2 for its overall activation z-scores (3.5 vs 2.5). IPA, Ingenuity pathway analysis. (JPG 3319 kb) Additional file 9: Table S6. IPA analysis results obtained in case 1. IPA analysis results obtained in case 2. (XLS 232 kb)

## Abbreviations

AML: acute myeloid leukemia
FISH: fluorescence in situ hybridization
IPA: Ingenuity pathway analysis
MDS: myelodysplastic syndromes
PCR: polymerase chain reaction
RT-PCR: reverse transcription polymerase chain reaction
RT-qPCR: reverse transcription quantitative polymerase chain reaction
