# Supplementary material for: t(15;21) translocations leading to the concurrent downregulation of RUNX1 and its transcription factor partner genes SIN3A and TCF12 in myeloid disorders
Source: Mol Cancer. 2015 Dec 16;14:211. doi: 10.1186/s12943-015-0484-0 (PMC4681058; doi:10.1186/s12943-015-0484-0)
Supplement: Additional file 1: Table S1. — Cytogenetic and clinical features of the patients included in the study. (DOCX 8 kb) [file 12943_2015_484_MOESM1_ESM.docx]

**Table S1**: Cytogenetic and clinical features of the patients included in the study.

* Karyotype at diagnosis: 46,XX,del(9)(q13q22)[21/34]/46,XX[13/34]

** Karyotype at diagnosis: 47,XX,+9,der(20)t(1;20)(q21;p13)[9]/47,XX,+9,t(10;14)(p15;q11q31),der(20)t(1;20)(q21;p13)[2]/46,XX[1]

| **Case no.** | **Age/sex** | **Diagnosis** | **Karyotype** | **Investigated sample** | **Outcome** |
| --- | --- | --- | --- | --- | --- |
| 1 | 85/M | CMML | 46,XY,t(15;21)(q22?;q22?),-17,+der(17?)[10] | Onset | Supportive therapy with red blood cells, died within few months after diagnosis. |
| 2 | 45/F | AML M2 | 46,XX,t(15;21)(q21;q22)[5/32]/  46,XX,t(5;15)(q35;q13)[3/32]/  46,XX,t(V;21)(V;q22)[5/32]/46,XX[19/32] | Progression (six months after diagnosis*) | Progression after a six-months treatment according to SAKK/HOVON 42 protocol Arm A, followed by stem cell transplantation and complete remission after eight years. |
| 3 | 76/M | t-AML | 46,XY,del(12)(p11.2p12~13)[15/23]/  46,idem, t(15;21)(q21;q22)[6/23]/46,XY[2/23] | Onset | Failure after azacytidine treatment, dead of a septic shock after on cycle of azacytidine treatment. |
| 4 | 41/F | aCML | 47,XX,+9,t(15;21)(q21;q22),der(20)t(1;20)(q21;p13)[10] | Progression (eight years after diagnosis**) | Failure after nine years of various treatments: hydroxyurea and/or interferon for the first six years; busulfan, hydroxyurea, imatinib and intravenous Cytarabine in the last three years. Died due to a massive blast crisis nine years after diagnosis. |
