# Supplementary material for: t(15;21) translocations leading to the concurrent downregulation of RUNX1 and its transcription factor partner genes SIN3A and TCF12 in myeloid disorders
Source: Mol Cancer. 2015 Dec 16;14:211. doi: 10.1186/s12943-015-0484-0 (PMC4681058; doi:10.1186/s12943-015-0484-0)
Supplement: Additional file 2: Table S2. — FISH results obtained with BAC and fosmid probes in all the cases included in the study. (DOCX 125 kb) [file 12943_2015_484_MOESM2_ESM.docx]

**Table S2.** FISH results obtained with BAC and fosmid probes in all the cases included in the study.

| **PROBE NAME** | **MAP POSITION (GRCh37/hg19)** | **FISH RESULTS** |
| --- | --- | --- |
| **CASE 1** | | |
| RP11-941F15 | chr15:74,155,812-74,332,998 | 15, der(15) |
| RP11-10O17 | chr15:74,699,375-74,863,578 | 15, der(15) |
| G248P80492A4 | chr15:74,736,938-74,776,448 | 15, der(15) |
| G248P8834G10 | chr15:74,750,426-74,796,634 | 15, der(15) |
| G248P82315G1 | chr15:74,783,796-74,822,345 | 15, der(15) |
| RP11-958O2 | chr15:74,816,744-75,032,060 | 15 |
| G248P88977E8 | chr15:74,823,227-74,865,372 | 15 |
| RP11-581I24 | chr15:74,981,780-75,166,298 | 15 |
| RP11-663D24 | chr15:75,234,395-75,443,209 | 15 |
| G248P88322E3 | chr15:75,634,004-75,672,379 | 15 |
| G248P89858D10 | chr15:75,677,802-75,718,262 | 15, der(21) |
| G248P89994B11 | chr15:75,691,916-75,731,720 | 15, der(21) |
| G248P87955C12 | chr15:75,708,634-75,750,089 | 15, der(21) |
| G248P89638D11 | chr15:75,724,555-75,766,287 | 15, der(21) |
| RP11-875K24 | chr15:75,686,521-75,899,896 | 15, der(21) |
| RP11-162F14 | chr15:76,169,570-76,321,061 | 15, der(21) |
| RP11-659C9 | chr15:76,274,407-76,484,999 | 15, der(21) |
| RP11-625F11 | chr15:76,501,726-76,653,607 | 15, der(21) |
| RP11-691L10 | chr15:76,673,374-76,849,870 | 15, der(21) |
| RP11-96H23 | chr15:79,216,315-79,397,802 | 15, der(21) |
| RP11-89K11 | chr15:102,162,098-102,308,889 | 15, der(21) |
| RP11-299D9 | chr21: 36,141,211-36,280,531 | 21, der(21), der(15) |
| G248P88990G6 | chr21:36,147,772-36,184,429 | 21, der(21) |
| G248P80218C10 | chr21:36,159,062-36,202,107 | 21, der(21), der(15) |
| G248P85591F4 | chr21:36,171,229-36,210,578 | 21, der(21), der(15) |
| RP11-483M4 | chr21: 36,287,033-36,459,143 | 21, der(15) |
| **CASES 2 AND 3** | | |
| RP11-829L24 | chr15:42,051,348-42,231,529 | 15, der(15) |
| RP11-121N6 | chr15:44,730,858-44,879,213 | 15,der(15) |
| RP11-140M10 | chr15:50,457,043-50,619,701 | 15,der(15) |
| RP11-133J10 | chr15:53,752,902-53,935,303 | 15,der(15) |
| RP11-80N16 | chr15:55,748,331-55,903,311 | 15,der(15) |
| RP11-976I14 | chr15:56,724,165-56,884,707 | 15,der(15) |
| RP11-278A12 | chr15:57,054,594-57,216,487 | 15,der(15) |
| RP11-92P3 | chr15:57,216,125-57,413,919 | 15;der(21) |
| RP11-236P11 | chr15:64,579,850-64,723,351 | 15;der(21) |
| RP11-875K24 | chr15:75686521-75899896 | 15;der(21) |
| RP11-378B5 | chr15:91,673,519-91,849,468 | 15;der(21) |
| RP11-299D9 | chr21: 36,141,211-36,280,531 | 21, der(21), der(15) |
| G248P88990G6 | chr21:36,147,772-36,184,429 | 21, der(21) |
| G248P80218C10 | chr21:36,159,062-36,202,107 | 21, der(21), der(15) |
| G248P85591F4 | chr21:36,171,229-36,210,578 | 21, der(21), der(15) |
| RP11-483M4 | chr21: 36,287,033-36,459,143 | 21, der(15) |
| **CASE 4** | | |
| RP11-299D9 | chr21: 36,141,211-36,280,531 | 21, der(21) [faint signal] |
| G248P88990G6 | chr21:36,147,772-36,184,429 | 21, der(21) |
| G248P80218C10 | chr21:36,159,062-36,202,107 | 21, der(21) |
| G248P85591F4 | chr21:36,171,229-36,210,578 | 21, der(21) |
| G248P87919E12 | chr21:36,214,863-36,253,274 | 21 |
| G248P88378B1 | chr21:36,256,821-36,296,435 | 21 |
| RP11-483M4 | chr21: 36,287,033-36,459,143 | 21 |
| G248P88558C9 | chr21:36,304,978-36,342,761 | 21 |
| RP11-384N13 | chr21:36,592,109-36,753,937 | 21 |
| RP11-60N19 | chr21:36,701,580-36,847,997 | 21 |
| RP11-662O10 | chr21:36,785,367-36,948,113 | 21, der(15) [faint signal] |
| RP11-299P9 | chr21:36,896,525-37,088,598 | 21, der(15) |
| RP11-714H12 | chr21:37,039,716-37,207,890 | 21, der(15) |
| RP11-476D17 | chr21:39,682,968-39,863,671 | 21, der(15) |
| RP11-162F19 | chr21:46,532,032-46,691,772 | 21, der(15) |
| RP11-998E11 | chr15:49,088,667-49,248,812 | 15, der(15) |
| RP11-629G2 | chr15:56,362,561-56,544,874 | 15, der(15) |
| RP11-89A6 | chr15:56,596,263-56,751,714 | 15, der(15) |
| RP11-71A21 | chr15:56,869,200-57,026,613 | 15, der(15) |
| RP11-31H5 | chr15:56,978,858-57,164,253 | 15, der(15) |
| RP11-179M20 | chr15:57,088,794-57,248,860 | 15 |
| RP11-92P3 | chr15:57,216,125-57,413,919 | 15, der(21), der(15) |
| RP11-11I7 | chr15:58,929,522-59,088,920 | 15, der(21) |
| RP11-265J2 | chr15:59,323,248-59,479,332 | 15, der(21) |
| RP11-314H3 | chr15:60,743,226-60,901,257 | 15, der(21) |
| RP11-410D7 | chr15:62,078,709-62,225,924 | 15, der(21) |
| RP11-45J10 | chr15:63,792,871-63,949,711 | 15, der(21) |
| RP11-196N10 | chr15:64,639,741-64,812,979 | 15, der(21) |
| RP11-726C22 | chr15:68,509,222-68,680,656 | 15, der(21) |
| RP11-119K8 | chr15:72,417,542-72,605,097 | 15, der(21) |
| RP11-941F15 | chr15:74,155,812-74,332,998 | 15, der(21) |
| G248P89858D10 | chr15:75,677,802-75,718,262 | 15, der(21) |
| G248P87955C12 | chr15:75,708,634-75,750,089 | 15, der(21) |
