# Supplementary material for: t(15;21) translocations leading to the concurrent downregulation of RUNX1 and its transcription factor partner genes SIN3A and TCF12 in myeloid disorders
Source: Mol Cancer. 2015 Dec 16;14:211. doi: 10.1186/s12943-015-0484-0 (PMC4681058; doi:10.1186/s12943-015-0484-0)
Supplement: Additional file 4: Table S3. — SNP array results [EMBL: E-MTAB-3782] obtained in case 1. (DOCX 123 kb) [file 12943_2015_484_MOESM4_ESM.docx]

**Table S3.** SNP array results [EMBL: E-MTAB-3782] obtained in case 1.

| **Chr** | **Cytoband Start** | **Cytoband End** | **Min** | **Max** | **Size (kbp)** | **Marker Count** | **CN State** | **Type** |
| --- | --- | --- | --- | --- | --- | --- | --- | --- |
| 2 | q11.2 | q11.2 | 97765096 | 97878057 | 113 | 36 | 3 | Gain |
| 6 | p12.1 | p12.1 | 54664689 | 55049020 | 384 | 304 | 1 | Loss |
| 6 | p22.1 | p22.1 | 29837071 | 29897260 | 60 | 39 | 3 | Gain |
| 6 | p23 | p22.3 | 14217300 | 15593600 | 1376 | 1134 | 0 | Loss |
| 6 | p25.3 | p25.3 | 302183 | 380993 | 79 | 135 | 3 | Gain |
| 8 | p11.22 | p11.22 | 39247097 | 39386952 | 140 | 76 | 0 | Loss |
| 11 | q11 | q11 | 55374018 | 55452996 | 79 | 100 | 0 | Loss |
| 12 | p13.2 | p13.1 | 10854820 | 13563547 | 2709 | 3179 | 1 | Loss |
| 12 | q23.1 | q23.1 | 99791778 | 99798721 | 7 | 32 | 1 | Loss |
| 14 | q24.3 | q24.3 | 73995760 | 74020966 | 25 | 26 | 4 | Gain |
| 14 | q32.33 | q32.33 | 106074223 | 106952842 | 879 | 298 | 3 | Gain |
| 15* | q24.1 | q24.2 | 74800104 | 75707817 | 908 | 656 | 1 | Loss |
| 16 | p11.2 | p11.1 | 34471297 | 34755816 | 285 | 172 | 3 | Gain |
| 17 | p11.2 | p11.2 | 19244887 | 19318214 | 73 | 64 | 1 | Loss |
| 17 | q21.31 | q21.31 | 44212823 | 44246372 | 34 | 48 | 3 | Gain |
| 17 | q25.3 | q25.3 | 79239746 | 79437705 | 198 | 116 | 1 | Loss |
| 18 | q12.1 | q12.1 | 27458746 | 28098609 | 640 | 436 | 3 | Gain |
| 22 | q11.22 | q11.22 | 23121367 | 23258369 | 137 | 88 | 3 | Gain |
| 22 | q13.2 | q13.2 | 42523209 | 42540108 | 17 | 26 | 3 | Gain |
| X | p11.4 | p11.4 | 39051752 | 40407059 | 1355 | 840 | 0 | Loss |
| X | p22.33 | p22.33 | 2680070 | 2703633 | 24 | 43 | 4 | Gain |
| X | p22.33 | p22.33 | 2302148 | 2303322 | 1 | 28 | 4 | Gain |

* The deleted region on der(15) is highlighted in red.
