# Supplementary material for: t(15;21) translocations leading to the concurrent downregulation of RUNX1 and its transcription factor partner genes SIN3A and TCF12 in myeloid disorders
Source: Mol Cancer. 2015 Dec 16;14:211. doi: 10.1186/s12943-015-0484-0 (PMC4681058; doi:10.1186/s12943-015-0484-0)
Supplement: Additional file 5: Table S4. — List of primers used for RT-PCR, RT-qPCR and Sanger sequencing. (DOC 91 kb) [file 12943_2015_484_MOESM5_ESM.doc]

**Table S4.** List of primers used for RT-PCR, RT-qPCR and Sanger sequencing.

| **PRIMER NAME** | **SEQUENCE (5'→3')** | **MAP POSITION (hg19)** |
| --- | --- | --- |
| SIN3A_E1_F | CCCCTCCTGTGTATGAAGCA | chr15:75,722,588-75,722,607 |
| SIN3A_E1_R | ACTGAATTCCCGTAGCTGACT | chr15:75,722,551-75,722,571 |
| SIN3A_E2_F | TTTCAGCCATGCCACAGAG | chr15:75,715,142-75,715,163 |
| SIN3A_E4_F | TCCCAGCTATTCAAAGGCCA | chr15:75,705,336-75,705,355 |
| SIN3A_E4_R | TGAAGGCTGGGAAGGATGTT | chr15:75,705,167-75,705,186 |
| SIN3A_E5_F | CACTTCCACCGTATGCATCC | chr15:75,704,019-75,704,038 |
| SIN3A_E5_R | GTAGATGTCTGGTTGGCCCT | chr15:75,703,866-75,703,885 |
| RUNX1_E5_F | AGCTTCACTCTGACCATCACT | chr21:36,231,850-36,231,870 |
| RUNX1_E5_R | TGATGGCTCTGTGGTAGGTG | chr21:36,231,804-36,231,823 |
| UBL7_AS1_1F | CTGCTATGGACACAGATGGC | chr15:74,773,407-74,773,426 |
| UBL7_AS1_1R | GCCACCACTCTAGGCTCTAC | chr15:74,773,497-74,773,516 |
| SIN3A_I3_F | TTGCGGATCTTCTCTGGGTT | chr15:75,707,835-75,707,854 |
| SIN3A_I3_R | CAGCGCTTACATCCTTCCAC | chr15:75,707,749-75,707,768 |
| SIN3A_5UTR_F | ATGAGCACAGAATGAAGCGG | chr15:75,722,727-75,722,746 |
| RUNX1_UTR_R | GACCTACAGCGAGATCCTGG | chr21:36,164,273-36,164,292 |
| RUNX1_I6_F | CCTTGAAAGTGGAGGCTACA | chr21:36,202,011-36,202,030 |
| RUNX1_I6_R | CTGGAAAGGCTCTCATACCCT | chr21:36,201,832-36,201,852 |
| UBL7_CHIM_F | AGGGTGCATTTTCAGGAGGA | chr21:36,421,201-36,421,220 |
| RUNX1_I6_1R | GAGGAGGGGAAAGGAAAGCT | chr21:36,191,271-36,191,290 |
| RUNX1_I6_1F | ACTGGAGCATGGCAGTAACT | chr21:36,191,676-36,191,695 |
| RUNX1_I6_2R | CCCTTGTGAGAATCCTGGGT | chr21:36,185,777-36,185,796 |
| RUNX1_I6_2F | TGTCTCGGAGGTTGCTTTCT | chr21:36,186,107-36,186,126 |
| RUNX1_I6_3R | AGCTGGTGTAATGCCCTCTT | chr21:36,179,938-36,179,957 |
| RUNX1_I6_3F | TTCTTACTTGTGCTGTGCCG | chr21:36,180,286-36,180,305 |
| RUNX1_I6_4F | TGAGTCCTAGTTACCCGGGA | chr21:36,198,856-36,198,875 |
| RUNX1_I6_4R | AGCAAACGGGTCATGAGGAT | chr21:36,198,492-36,198,511 |
| UBL7_I1_1R | GAGCAGAGATCACGCAACTG | chr15:74759337-74759356 |
| UBL7_I1_1F | TGCTCTTTTCACCAGGTTGC | chr15:74,758,861-74,758,880 |
| RUNX1_I6_5F | AGAAATTCAGGCCCACTCGA | chr21:36,197,110-36,197,129 |
| RUNX1_I6_5R | CCATTTAGGGGAGCGTGAAC | chr21:36,196,488-36,196,507 |
| RUNX1_I6_6F | TTGCCAGTTCCTGCTATGGA | chr21:36,195,390-36,195,409 |
| RUNX1_I6_6R | GGACTGGGACTTGGTGGTAA | chr21:36,194,487-36,194,506 |
| UBL7_AS1_RIG | TGCTACCCTGAACCACATCT | chr15:74,760,201-74,760,220 |
| SIN3A_I3A_R | TGACCTGTGATCCTTCCCAC | chr15:75,708,545-75,708,564 |
| SIN3A_I3A_F | AGCAGTGTTGTTATCGCACA | chr15:75,708,502-75,708,521 |
| RUNX1_I6A_F | GGTCTCCTGTAGCTGGTTGT | chr21:36,192,434-36,192,453 |
| RUNX1_I6A_R | GTCTCGAACTCCTGACCACA | chr21:36,192,730-36,192,751 |
| RUNX1_TER_R | GTCGACTGGAAAGTTCTGCA | chr21:36,171,604-36,171,623 |
| RUNX1_E9R | CGTCGGGGAGTAGGTGAAG | chr21:36,164,800-36,164,820 |
| RUNX1_E91R | CTCTGGTTCGGGAGGCTG | chr21:36,164,524-36,164,544 |
| RUNX1_ALT_R | AAGGTGCTGATCTTGGGTGA | chr21:36,260,725-36,260,744 |
| RUNX1_ALT_F | TTCCAGTGTTCTTGGGTGGT | chr21:36,260,846-36,260,865 |
| RUNX1_E4_AL | GCACTGAGCGTTTTGACAGA | chr21:36,260,182-36,260,201 |
| RUNX1_ALT_R | GCTTCAGTCCCGAAAAGCAA | chr21:36,259,929-36,259,948 |
| RUNX1_EX9_2R | TGGTAGGAGGGCGAGCTG | chr21:36,164,663-36,164,682 |
| RUNX1_EX9_3R | CGGCAGGTAGGTGTGGTAG | chr21:36,164,728-36,164,747 |
| UBL-AS1E1F | GAAAACTGCTGTCCGTTGGT | chr15:74,753,915-74,753,934 |
| UBL-AS1E1R | TCGTCTGGTTCCTGGTATGC | chr15:74,753,995-74,754,014 |
| UBL7_AS1_I2_R | TTCAAGCATAAAGGCCAGAAA | chr15:74,758,735-74,758,755 |
| RUNX1_I7_F | TGTGGAACAGCCATAACTGC | chr21:36,194,891-36,194,910 |
| RUNX1_I7A_F | TCTCCCAGCCCTGTAAATTG | chr21:36,195,407-36,195,426 |
| UBL7_AS1_I2_F | ATCTCCACCCCAGGTTCAAG | chr15:74,758,411-74,758,430 |
| RUNX1_I7_R | TAAAACGGGGAAGTGGGTGA | chr21:36,194,787-36,194,806 |
| RUNX1_I7A_R | AAGAGGAACTGGCACAGCAT | chr21:36,195,293-36,195,312 |
| UBL7_AS1A_R | TAGGCAGGAGAGGAAGGAGT | chr15:74,754,706-74,754,725 |
| UBL7_AS1B_R | AAGACGTGTGACATTGGCAC | chr15:74,755,459-74,755,478 |
| UBL7_AS1_I1C | AGTCTGCCCCATGATCCAAA | chr15:74,756,674-74,756,693 |
| UBL7_AS1_F | GCAGGATCTCAGAAGGCCTA | chr15:74,757,166-74,757,185 |
| UBL7_ASI1C_F | GGTGGAGGCTGATCATCTCT | chr15:74,756,067-74,756,086 |
| RUNX1_I7B_F | ATGAGGGACACGAACATGGT | chr21:36,195,150-36,195,169 |
| RUNX1_I7C | AGGACTACATATGTCCAGTTGCT | chr21:36,195,258-36,195,280 |
| SIN3A_EX1_F | ATGCAGTCAGCTACGGGAAT | chr15:75,722,556-75,722,575 |
| SIN3A_EX2_R | TGATGGCTGCTATGAACTGC | chr15:75,715,097-75,715,116 |
| SIN3A_EX2-3_F | AGAGGCTGAAGGTGGAGGAT | chr14:54,630,824-54,630,843 |
| SIN3A_EX3_R | GCCTTTGAATAGCTGGGACA | chr15:75,705,338-75,705,357 |
| SIN3A 3'UTR_F | TTTGAGGCATGTTTGGTGTT | chr15:75,712,593-75,712,612 |
| SIN3A 3'UTR_R | CCCCGAATCTCCTGAAGTCT | chr15:75,712,478-75,712,497 |
| RUNX1EX8 | CCCACCATGGAGAACTGGTA | chr21:36,164,616-36,164,635 |
| RUNX1_E7F | ACAAGGCAGATCCAACCATC | chr21:36,171,738-36,171,757 |
| RUNX1_E7R | ACAGAAGGAGAGGCAATGGA | chr21:36,171,678-36,171,697 |
| RUNX1EX1F | GGCTTCAGACAGCATATTTGAG | chr21:36,421,173-36,421,194 |
| RUNX1E1R | AAGCACTGTGGGTACGAAGG | chr21:36,421,147-36,421,166 |
| RUNX1E5NEWF | GGAAAAGCTTCACTCTGACCA | chr21:36,231,855-36,231,875 |
| TCF12_E1_F | ATGAATCCCCAGCAACAACG | chr15:57,212,112-57,212,131 |
| TCF12_E1_R | AGCTCCTTGTCGGTCCCTAT | chr15:57,212,142-57,212,161 |
| TCF12_E2_F | TTTCCCCACCTGTTAATAGTGG | chr15:57,213,228-57,213,249 |
| TCF12_E2_R | CCACTGAATTGACTGCTTCCC | chr15:57,213,271-57,213,291 |
| TCF12_E4_F | GGTTTTACAGACAGCCCTCA | chr15:57,383,987-57,384,006 |
| TCF12_E4_R | TCATGGGCTCCTAATCGACT | chr15:57,384,029-57,384,048 |
| TCF12_E6_F | CAAGATCTGGGGCTTGGGA | chr15:57,484,370-57,484,390 |
| TCF12_E6_R | GCAGAGTCATGGAGTGGTCT | chr15:57,484,464-57,484,483 |
