# Supplementary figures and images for: t(15;21) translocations leading to the concurrent downregulation of RUNX1 and its transcription factor partner genes SIN3A and TCF12 in myeloid disorders
Source: Mol Cancer. 2015 Dec 16;14:211. doi: 10.1186/s12943-015-0484-0 (PMC4681058; doi:10.1186/s12943-015-0484-0)

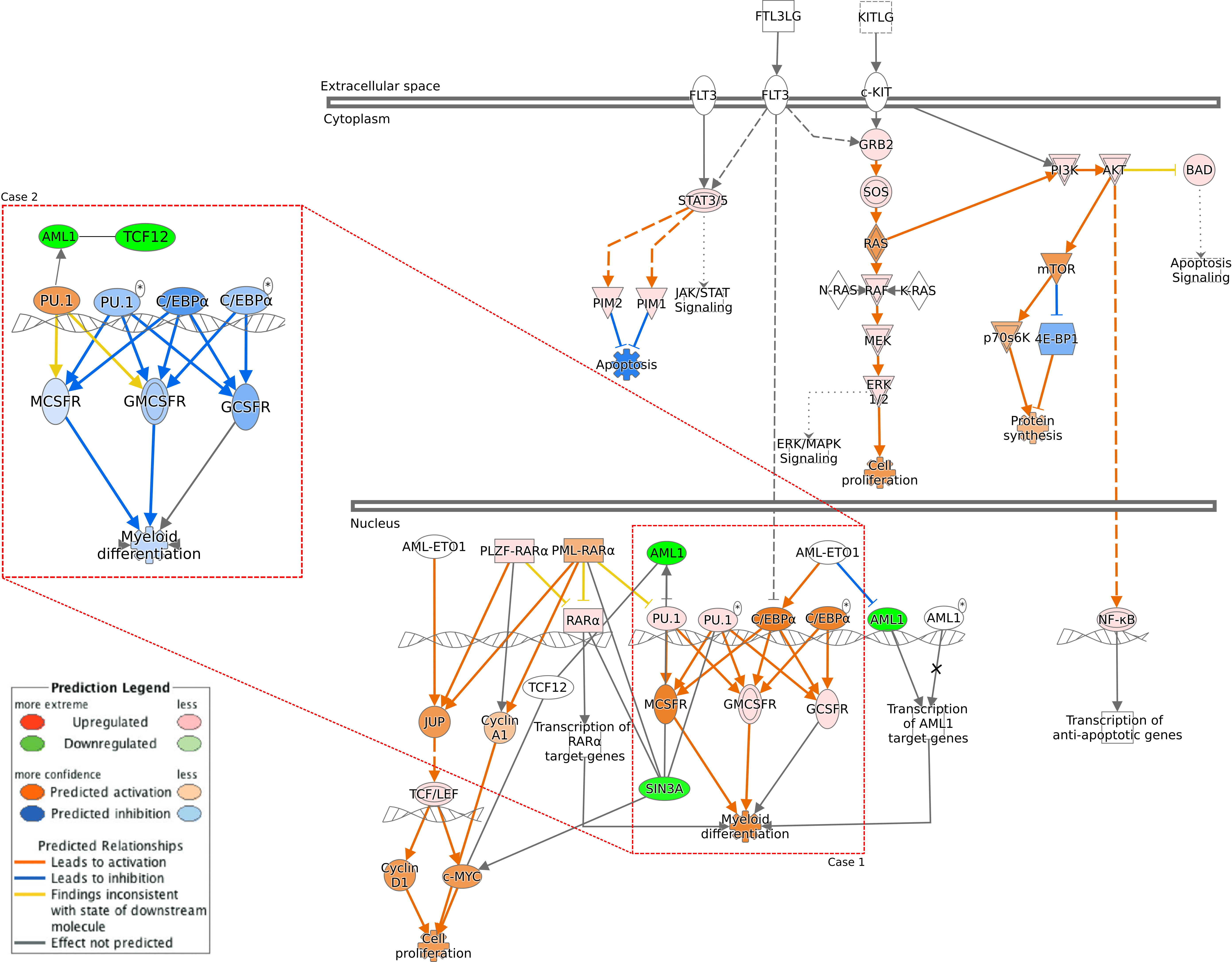

Supplement: Additional file 8: Figure S3. — The IPA acute myeloid leukemia signaling pathway for cases 1 and 2. Orange and blue glyphs indicate genes with predicted enhanced or reduced activity, respectively. Green symbols represent downregulated genes. Equally, orange and blue toothed wheels stand for activated or inhibited biological processes. Case 1 differs from case 2 for its overall activation z-scores (3.5 vs 2.5). IPA, Ingenuity pathway analysis. (JPG 3319 kb) [file 12943_2015_484_MOESM8_ESM.jpg]
